# Adjuvant modern radiotherapy in resected pN2 NSCLC patients: results from a multicentre retrospective analysis on acute and late toxicity on behalf of AIRO thoracic oncology study group: the RAC-TAC study

**DOI:** 10.1007/s11547-024-01885-w

**Published:** 2024-08-31

**Authors:** Valerio Nardone, Alessio Bruni, Davide Franceschini, Beatrice Marini, Stefano Vagge, Patrizia Ciammella, Matteo Sepulcri, Anna Cappelli, Elisa D’Angelo, Giuseppina De Marco, Antonio Angrisani, Mattia Manetta, Melissa Scricciolo, Cesare Guida, Dario Aiello, Paolo Borghetti, Salvatore Cappabianca

**Affiliations:** 1 Department of Precision Medicine, University of Campania “L. Vanvitelli”, Naples, Italy; 2 Radiotherapy Unit, Department of Oncology and Hematology, University Hospital of Modena, Modena, Italy; 3 Department of Radiotherapy and Radiosurgery, IRCCS Humanitas Research Hospital, Milan, Italy; 4 Department of Biomedical Sciences, Humanitas University, Milan, Italy; 5 Radiation Oncology Department, E.O. Ospedali Galliera, Genoa, Italy; 6 Radiation Oncology Unit, Azienda USL-IRCCS Di Reggio Emilia, 42123 Reggio Emilia, Italy; 7 Radiotherapy, Veneto Institute of Oncology IOV-IRCCS, Padua, Italy; 8 Radiotherapy Unit, University Hospital of Modena, Modena, Italy; 9 Radiation Oncology Department, Bellaria Hospital, AUSL of Bologna, Bologna, Italy; 10 Radiation Oncology Department, ASST Spedali Civili and University of Brescia, Brescia, Italy; 11 Azienda ULSS 3 Venezia, UOC Radioterapia Oncologica, Venezia, Italy; 12 Radiotherapy Unit, ASL Napoli 1 Centro, Ospedale del Mare, Naples, Italy; 13 Radiation Oncology, Casa Di Cura Macchiarella, Palermo, Italy; 14 Radiation Oncology, Oncology Institute of Southern Switzerland, EOC, Bellinzona, Switzerland

**Keywords:** NSCLC, PORT, Post-operative radiotherapy, Adjuvant therapy, Lung cancer, Radiotherapy

## Abstract

**Background:**

Recently, the PORT-C and LUNG-ART trials, which evaluated the role of postoperative radiation therapy (PORT), have significantly altered the treatment landscape for NSCLC pN2 patients who previously underwent surgery. In response, the Italian Association of Radiotherapy and Oncology Thoracic Oncology study group has initiated an observational multicenter trial to assess both acute and late toxicities of PORT in pN2 NSCLC patients treated with modern techniques.

**Methods:**

Data on NSCLC patients submitted to PORT after radical surgery treated between 2015 and 2020 in six Italian Centers were collected. Heart, lung, and esophageal acute and late toxicities have been retrospectively analyzed and related to radiation therapy dosimetric parameters. Furthermore, loco-regional control, distant metastasis and overall survival have been analyzed.

**Results:**

A total of 212 patients with a median age of 68 years from six different centers were included in this analysis (142 males and 70 females). Prior to undergoing PORT, 96 patients (45.8%) had a history of heart disease, 110 patients (51.9%) had hypertension, and 51 patients (24%) had COPD.

Acute toxicity was observed in 147 patients (69.3%), with lung toxicity occurring in 93 patients (G1 in 70 patients, G2 in 17 patients, and G3 in 4 patients), esophageal toxicity in 114 patients (G1 in 89 patients, G2 in 23 patients, and G3 in 1 patient), and cardiac toxicity in 4 patients (G1 in 2 patients and G3 in 2 patients). Late side effects were found in 60 patients (28.3%), predominantly involving the lungs (51 patients: 32 G1, 11 G2, and 1 G3) and the esophagus (11 patients: 8 G1 and 3 G2), with no reported late cardiac side effects.

Various clinical and dosimetric parameters were found to correlate with both acute and chronic toxicities. Over a median follow-up period of 54 months, 48 patients (22.6%) showed locoregional disease relapse, 106 patients (50%) developed distant metastases, and 66 patients (31.1%) died.

**Conclusions:**

RAC-TAC retrospective multicentric study showed the low toxicity of PORT when advanced technology is used. At the same time, it’s noteworthy to underline that 50% of the patients develop distant recurrences in the follow up.

## Introduction

The landscape of treatments for patients with pN2 non-small cell lung cancer (NSCLC) who have undergone surgery has been significantly transformed by recent developments in postoperative radiation therapy (PORT), as demonstrated by studies such as LUNG-ART and PORT-C [1, 2]. Clinical outcomes from both trials, particularly regarding overall survival (OS) (which did not reach the statistical significance in either study) and concerns related to radiotherapy (RT) toxicity, reduced the indication for PORT, especially among patients without risk factors [3].

Nonetheless, in our point of view, it is essential to scrutinize the results of these studies with a more balanced perspective. Notably, it is worth highlighting that the toxicity concerns observed in the Lung-ART trial were not substantiated by the findings in the PORT-C trial, which predominantly used advanced more sophisticated techniques (89.3% in PORT-C versus 11% in Lung-ART) such as volumetric modulated arc RT (VMAT) or intensity modulated RT (IMRT). Nevertheless, it remains critical to acknowledge that neither study demonstrated an advantage in OS and the substantial occurrence of deaths not related to cancer in the PORT arms of both trials underscores the necessity for a thorough assessment of the benefit/risk ratio associated with adjuvant approaches.

We believe that in the next future we will be able to correctly identify the pN2 patients that deserve adjuvant systemic therapies [4]. Among these strategies, RT should be performed in a proper way not to jeopardize the important advantage in mediastinal relapse (25% in PORT versus 46% in control group in the LUNG-ART trial).

In this context, the Italian Association of Radiotherapy and Oncology (AIRO) Thoracic Oncology Cancer study group has recently designed a multicenter retrospective observational study to assess both acute and late toxicities in NSCLC patients who received PORT using IMRT or VMAT techniques. The primary focus is on RT-related side effects. Secondary objectives include local control, distant control, overall survival, and dosimetry assessments of organs at risk (heart, lungs, and esophagus). This comprehensive approach, which includes toxicity assessment, dosimetry analysis, and clinical outcome evaluation, enhances our understanding of the treatment landscape and paves the way for further advancements in patient care.

## Methods

### Study design and population

The study adopted an observational retrospective design. Patients eligible for inclusion had histologically confirmed NSCLC. All patients underwent surgery with mediastinal exploration, which revealed mediastinal involvement (pN2) and subsequently received PORT using IMRT or VMAT techniques.

Exclusion criteria were: presence of documented metastases in a different lung lobe, pleural or pericardial effusion, history of prior thoracic RT, clinical progression during post-operative chemotherapy, and recent (within the last 6 months) severe cardiac or pulmonary diseases.

### Data collection and database

To facilitate data collection, a dedicated database was established. Data collection spanned from January 1, 2015, to December 31, 2020, aiming to accumulate a sufficiently large dataset for comprehensive analysis. The data collected included a wide range of information, such as demographic details (gender, age, and recruitment date), medical history (comorbidities and smoking habits), ECOG status, tumor features (staging, histology, biomarkers), treatment characteristics (type of RT, concurrent/sequential chemotherapy and treatment start/end dates) and therapy response assessed using RECIST criteria. Radiation and dosimetry parameters for RT (technique, dose per fraction, total dose, duration) encompassing cardiac (V5, V30, Mean Dose, DMAX [0.1 cc]), pulmonary (Mean Dose, V20, V30), and esophageal (Mean Dose, V50, V55) dosimetry, toxicity assessments based on the CTCAE 4.0 scale (acute and late) were analyzed. Furthermore, clinical outcomes, including overall survival (OS), progression-free survival (PFS), local (LC) and symptom control were also evaluated. Sample size determination relied on consecutively treated patients at participating centers. OS and PFS were calculated from diagnosis until death or disease progression, using Kaplan–Meier method. Acute toxicity during and at the end of RT treatment was analyzed descriptively, presenting raw incidence rates and severity. Late toxicity, assessed at least 6 months post-radiotherapy completion, underwent similar descriptive analysis, reporting raw incidence rates and severity.

### Statistical analysis

Data analysis involved a range of statistical techniques to examine the dataset. Descriptive statistics analyzed clinical and demographic variables, providing an overview of the study population. For the analysis of correlations, Pearson's correlation coefficient was used, allowing us to assess relationships between clinical factors, dosimetric parameters, and toxicity outcomes. Associations between categorical variables, such as comorbidities and toxicity, were explored using chi-squared or ANOVA where applicable. Logistic regression analysis was used for multivariate analysis. Statistical analyses used a two-sided approach and was considered as significant a p-value below 0.05. Data analysis was performed using IBM SPSS (IBM Corp, Armonk, NY, Version 23.0).

## Results

### Demographics

In this retrospective study, a total of 255 patients with a median age of 69 years (standard deviation 9.25, range 41–85 years) were included. One hundred sixty seven were males (65.5%) and 88 females (34.5%), among the participants, 42 (16.5%) were non-smokers, 166 (65.1%) former smokers and 44 (17.3%) current smokers. Clinical features of the entire cohort are summarized in Table 1.

**Table 1:** Clinical characteristics of the patients enrolled in the retrospective trial

Characteristic	Number/percentage
Total patients	255
Male	167 (65.5%)
Female	88 (34.5%)
Median age	69 years
*Smoking status*	
Non-smokers	42 (16.5%)
Former smokers	166 (65.1%)
Current smokers	44 (17.3%)
Unknown	3 (1.2%)
*ECOG performance status*	
ECOG 0	143 (56%)
ECOG 1	101 (39.6%)
ECOG 2	7 (2.7%)
ECOG 3	3 (1.2%)
*Hypertension*	
Yes	128 (50.2%)
No	126 (49.4%)
Missing	1 (0.4%)
*COPD (GOLD scale)*	
GOLD 0	137 (53.7%)
GOLD 1	28 (11%)
GOLD 2	25 (9.8%)
GOLD 3	2 (0.8%)
Unknown	63 (24.7%)
*Heart comorbidities*	
Yes	106 (41.6%)
No	145 (56.9%)
Missing	4 (1.6%)

Regarding the ECOG performance status, 143 patients (56%) had a score of 0, 101 (39.6%) had 1, 7 (2.7%) had 2, and 3 patients (1.2%) had a score of 3. Hypertension was present in 128 patients (50.2%), and the GOLD scale for chronic obstructive pulmonary disease (COPD) was distributed as follows: 137 (53.7%), 28 (11%), 25 (9.8%) and 2 (0.8%) patients had a score of 0, 1, 2 and 3, respectively.

The majority of patients were staged with 18 FDG PET/CT scan (228/255 patients, 88,7%), whereas brain MRI was used at baseline in only 30 patients (11,7%) and Endobronchial Ultrasound bronchoscopy for biopsy in 131 patients (51%).

### Surgery and chemotherapy treatments

After completion of the clinical staging, 62 patients (24.3%) were staged as cN0, 26 (10.2%) as cN1, 137 (53.7%) as cN2, and a single patient (0.4%) as cN3. Surgical procedures included lobectomy in 183 patients (71.8%), segmentectomy in 9 patients (3.5%), pneumonectomy in 27 patients (10.6%) and sleeve resection in 15 patients (5.9%). After pathological review following surgery, two patients were staged as pN0 (0,8%) and three patients as pN1 (1,2%), whereas the median number of positive nodes was 3 (range 1–20), and the median number of nodes removed was 11 (range 1–75).

Regarding margin status, 175 patients (68.6%) had negative margins (R0), while 12 (4.7%) were classified as R1 (9/12) and R2 (3/12).

Adjuvant therapy consisted of sequential chemo-radiotherapy in most cases (125 of 255 patients, 49%), with 41 of 255 patients (16.1%) undergoing concomitant adjuvant chemo-radiotherapy. Twenty-one patients (8.2%) received neoadjuvant chemotherapy and finally 68 (26.6%) did not undergo chemotherapy. In the adjuvant setting, the median number of chemotherapy cycles administered was 3 (range 1–8); the most common regimen was represented by cisplatin doublets (165 patients, 64,7%). Adjuvant therapies are summarized in Table 2.

**Table 2:** Summary of therapies delivered to the cohort of patients

haracteristic	Number/percentage
*Histological types*	
Adenocarcinoma	195 (76.5%)
Squamous cell carcinoma	47 (18.4%)
Other histological types	13 (5.1%)
*Surgical procedures*	
Lobectomy	183 (71.8%)
Segmentectomy	9 (3.5%)
Pneumonectomy	27 (10.6%)
Sleeve resection	15 (5.9%)
Median number of positive nodes	3 (range 1–20)
Median number of nodes removed	11 (range 1–75)
*Residual disease status after surgery*	
R0 (complete resection)	175 (68.6%)
R1 (microscopic residual disease)	9 (3.5%)
R2 (macroscopic residual disease)	3 (1.2%)
*Adjuvant chemotherapy*	
Sequential chemo-radiotherapy	125 (49%)
Concomitant adjuvant chemo-radiotherapy	41 (16.1%)
Neoadjuvant chemotherapy	21 (8.2%)
No chemotherapy	68 (26.6%)
Median number of chemotherapy cycles	3 (range 0–8)
*Radiotherapy technique*	
Tomotherapy	15%
Volumetric modulated arc therapy (VMAT)	74.8%
Intensity-modulated radiotherapy (IMRT)	Remaining patients
*Interruptions in radiotherapy*	
No interruptions	85.9%
Median interruption duration	7 days (range 2–40 days)

### Radiotherapy treatment

All patients received RT using advanced techniques, with 15% undergoing Tomotherapy, 74.8% VMAT and the remaining ones IMRT. RT was usually completed without any interruption, with only 14.1% experiencing a treatment suspension. The median interruption duration was 7 days (range 2–40 days).

Median RT doses received by the critical structures were as follows:

- Median doses to the heart for specific parameters were: V5 27%, V30 6%, mean and maximum dose were 7.83 Gy and 48 Gy;
- For bilateral lungs were: mean dose 10.1 Gy, V20:16% and V30: 8%;
- Median doses for the esophagus were 20 Gy as mean dose, 9.80% for V50, and no exposure for V55

### Toxicity

Regarding acute toxicities, 179 patients (70.2%) experienced different adverse events summarized in Table 3.

**Table 3:** Toxicity of post-operative radiotherapy (PORT)

Type of toxicity	Acute (number of patients)	Acute (G1/G2/G3) percentage	Chronic (number of patients)	Chronic (G1/G2/G3) percentage	Type of toxicity
Pulmonary toxicity	106 (41.6%)	G1: 83 (32.5%), G2: 19 (7.5%), G3: 4 (1.6%)	52 (20.4%)	G1: 38 (14.9%), G2: 13 (5.1%), G3: 1 (0.4%)	Pulmonary toxicity
Esophageal toxicity	137 (53.7%)	G1: 109 (42.7%), G2: 27 (10.6%), G3: 1 (0.4%)	12 (4.7%)	G1: 9 (3.5%), G2: 3 (1.2%)	Esophageal toxicity
Cardiac toxicity	4 (1.6%)	G1: 2 (0.8%), G3: 2 (0.8%)	1 (0.4%)	G3: 1 (0.4%)	Cardiac toxicity
Asthenia	63 (24.7%)	G1: 51 (20.0%), G2: 11 (4.3%), G3: 1 (0.4%)	18 (7.1%)	G1: 18 (7.1%)	Asthenia
Other acute toxicity*	19 (7.5%)	G1: 19 (7.5%)	–	–	Other acute toxicity

*Other acute toxicity include radiation dermatitis, nausea, and fever

Lung toxicities were observed in 106 patients being of grade 1 (G1) in 83 patients (46.4%), G2 in 19 (10.6%) and G3 in 4 patients (2.2%). Similarly, esophageal side effects occurred in 137 patients and were classified as G1, G2 and G3 in 109 (63.1%), 27 (15.6%) and one patient (0.6%), respectively. In terms of heart toxicity, cardiological events (pericarditis and atrial fibrillation) were observed only in 4 patients (2.2%), being G1 in 2 patients and G3 in 2 patients. Finally, 63 patients (27.9%) experienced asthenia, with severity categorized as G1 in 51 patients (22.6%), G2 in 11 (4.9%) and G3 in a single patient (0.4%). In 19 patients (7.5%) other types of acute toxicities were experienced, most of which were G1, including radiation dermatitis, nausea, and fever. It’s noteworthy to underline that the focus of this analysis was on toxicities specifically associated with radiotherapy, consequently, hematological toxicity hasn't been extensively addressed.

Moving to late toxicities, 71 patients (27.8%) developed lasting adverse effects that began at least six months after completing PORT. Chronic pulmonary toxicity was showed in 52 patients (20.4%), of whom 38 patients had G1 (14.9%), 13 patients G2 (5.1%) and only 1 patient G3 (0.4%). On the other hand, esophageal toxicity was noted in 12 patients (4.7%), being G1 in 9 patients (3.5%) and G2 in 3 patients (1.2%). Unfortunately, a single patient (0.4%) experienced severe (G3) late cardiac toxicity (heart failure). In addition, 18 patients (7.1%) exhibited other forms of chronic toxicity, predominantly G1.

### Outcomes

In the latest analysis 78 patients (30.6%) were free from disease, 19 (7.5%) had local failures, 36 (14.1%) developed metastatic disease and 19 (7.5%) had both local and distant recurrences, considering a median follow-up of 49 months. Seventy-one (27.8%) even died for disease progression, while 13 (5.1%) passed away due to other causes (not tumor related). Finally, 19 patients (7.5%) were lost to follow-up. Median overall survival was 51 months, with a standard deviation of 4.54 months (95% CI 42–59 months).

Furthermore, 135 patients (52.9%) developed distant dissemination, showing a median metastasis-free survival of 20 months, with a standard deviation of 4.1 months (95% CI 11–28 months). The most prevalent sites of metastatic disease were brain, affecting 26 out of 135 patients (19.3%), followed by bone in 22/135 (16.3%), lung in 16/135 (11.9%) and liver involvement in 4 patients (3.0%). Twenty patients (14.8%) developed metastasis in other sites, while synchronous multifocal metastasis were found in 47/135 patients (34.8%).

**Fig. 1:**
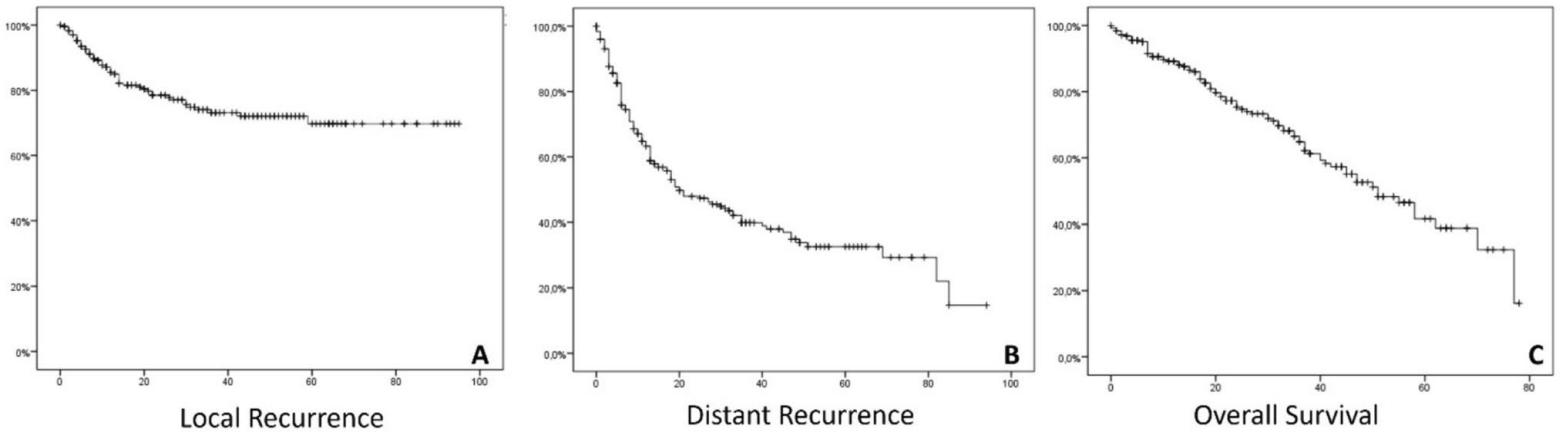
Survival curves for the three outcomes. Panel A: Local recurrence survival, showing that among the patients studied, 52 individuals (20.4%) experienced local recurrences, with a median progression-free survival that was not reached. The mean progression-free survival in this group was 71 months, with a standard deviation of 2.7 months (95% CI 66–77 months). Panel B: Distant recurrence survival, indicating that 135 patients (52.9%) developed distant disease during the follow-up period. The median progression-free survival for this group was 20 months, with a standard deviation of 4.1 months (95% CI 11–28 months). Panel C: Overall survival, demonstrating that 84 patients (32.9%) passed away during the follow-up period. The calculated median overall survival for the entire cohort was 51 months, with a standard deviation of 4.54 months (95% CI 42–59 months)

Finally, 52 patients (20.4%) experienced local recurrences, with a median PFS not reached, and a mean PFS of 71 months, with a standard deviation of 2.7 months (95% CI 66–77 months) (see Fig. 1).

### Correlation with toxicity

All patient characteristics (including demographics, tumor-related and treatment-related parameters) were collected in a centralized digital database trying to find a correlation with acute or late side effects as well as with clinical outcomes. At univariate analysis we observed associations between acute lung toxicity and several factors, such as preexisting cardiac disease (*p* = 0.030), number of removed lymph nodes (*p* = 0.042), heart Dmax (*p* = 0.031) and lung V20 (*p* = 0.011), whereas at multivariate analysis only preexisting cardiac disease (*p*:0,028) and heart-Dmax (*p*:0,043) remained significant. Furthermore, esophageal acute toxicity seems to be associated with gender (most common in females, 64,8% versus 48,5% in males, *p* = 0.013), number of removed lymph nodes (*p* = 0.006) and heart Dmax (*p* = 0.016), whereas at multivariate analysis only sex remained significant (*p*:0,018).

**Fig. 2:**
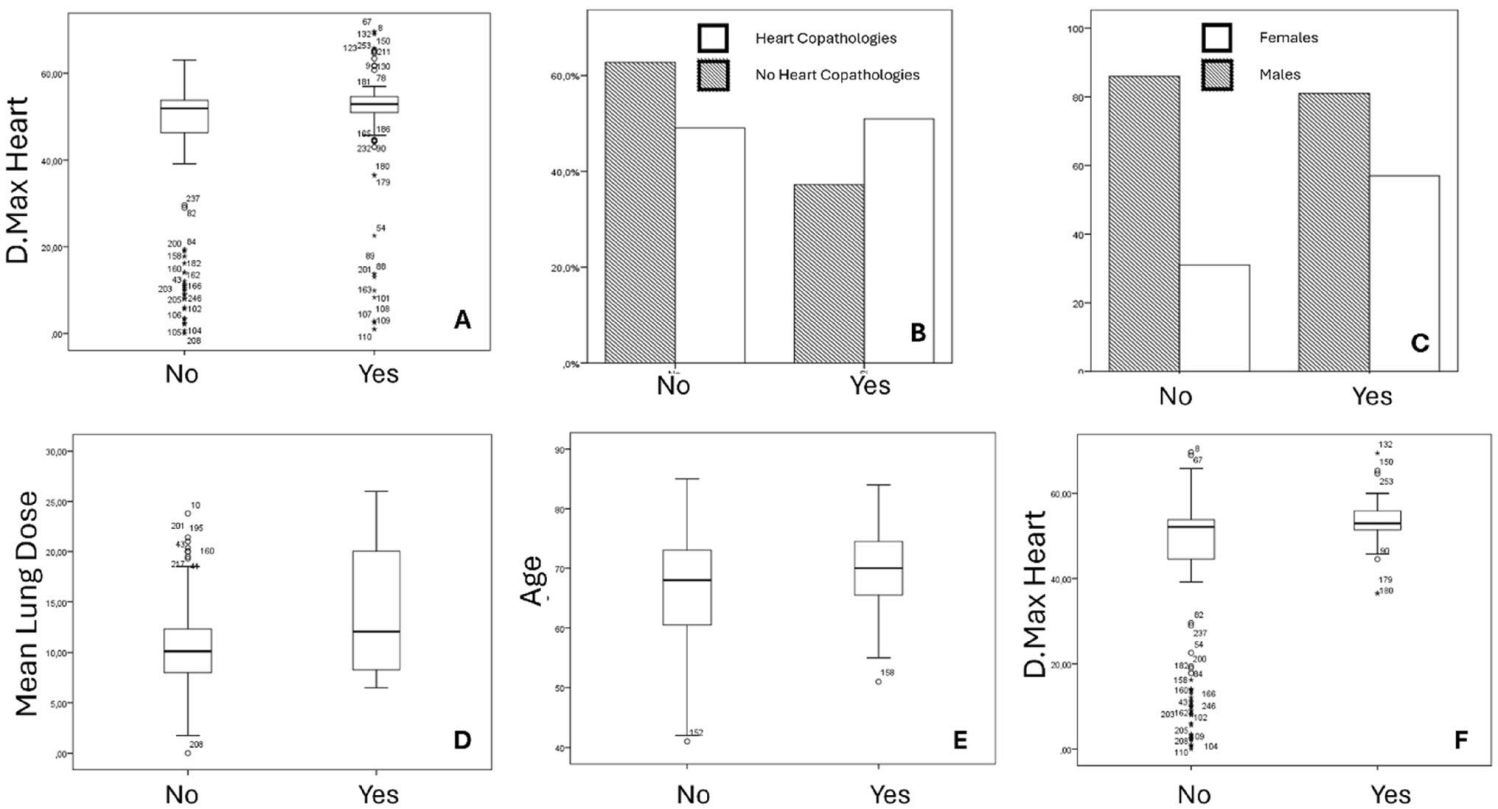
Correlation of significant factors with radiotherapy-related toxicities. The figure summarizes significant parameters identified through multivariate analysis in relation to radiotherapy-induced toxicities, with associated *p* values provided. *Panel A*: Comparison of maximum dose to the heart and acute lung toxicity (*p* = 0.043). *Panel B*: Relationship between heart comorbidities and acute lung toxicity (*p* = 0.028). *Panel C*: Gender-based analysis of esophageal toxicity (*p* = 0.018). *Panel D*: Mean lung dose and its correlation with acute heart toxicity (*p* = 0.048). *Panel E*: Correlation between age and acute asthenia (*p* = 0.014). *Panel F*: Relationship between acute and chronic toxicity and maximum dose to the heart (*p* = 0.002)

Acute cardiac toxicity was significantly associated with mean lung radiation dose (*p* = 0.017) and heart V5 (*p* = 0.043), but only mean lung radiation dose was significant at multivariate analysis (*p* = 0.048). Acute asthenia was found to be correlated with age at both univariate (*p* = 0.008) and multivariate analysis (*p*:0,014). Regarding chronic toxicities, lung toxicity was correlated with ECOG performance status (*p* = 0.010), preexisting cardiac disease (*p* = 0.033), COPD (*p* = 0.010), and heart Dmax (*p* < 0.001), whereas at multivariate analysis only heart Dmax (*p* = 0.002) remained significant. In Fig. 2 parameters that resulted significant at multivariate analysis are summarized.

## Discussion

In resected NSCLC pN2 the role of PORT has been a hot topic since 1998, when the PORT meta-analysis was published [5]. Although this analysis indicated a decrease in OS for patients receiving PORT, it has been criticized for utilizing outdated radiation therapy (RT) equipment and techniques, which can result in suboptimal dose distribution and increased radiation exposure to organs-at-risk, thereby worsening treatment-related mortality and limiting effectiveness [6, 7].

Due to the outdated nature of these data, its relevance to current practice was limited. However, there was still a lack of clear data to investigate the potential of PORT in this category of patients [8, 9]. In a monocentric retrospective analysis by Bruni, involving over 170 patients pN2 NSCLC, it was demonstrated that PORT might improve local control (*p* < 0.0009), with a 15% relapse rate in the PORT group compared to 32% in the non-PORT group [10], but without an impact in terms of distant metastasis and overall survival. The data on the use of PORT in conjunction with adjuvant chemotherapy are even more difficult to interpret, considering that the largest study was the ANITA secondary analysis [9].

Two recent phase III trials, LungART and PORT-C, found no benefits in terms of OS for PORT in stage III N2 NSCLC patients who had undergone surgery and adjuvant chemotherapy [1, 2]. The decision to use PORT, thus, involves considering several risk factors, such as resection margin, incomplete node dissection, extracapsular extension, multistation nodes, high ratio of positive nodes/excised nodes, impossibility to perform adjuvant chemotherapy or inadequate response to induction therapies, performance status and comorbidities [3, 11]. However, there is no unanimous consensus within the clinical community [3].

Future research should integrate clinical features and molecular biomarkers to identify the subsets of patients who may benefit or not from PORT [12, 13]. Upcoming studies should focus on enhancing the identification of high-risk patients for disease recurrence through the analysis of circulating tumor cells and the detection of postsurgical minimal residual disease.

Considering the above premises, it will be crucial to examine the impact of the radiation techniques to ensure the preservation of the advantages associated with enhanced local control. Indeed, in the last years the introduction of sophisticated techniques such as VMAT, IMRT or image guided radiotherapy in clinical practice has helped to improve the quality of radiation treatments in both the curative and palliative settings. The better knowledge and the longer experience in using these modern radiation therapy tools may increase the safety and the efficacy of the different treatments, particularly ameliorating the safety profile of RT [14]. Jairam conducted an analysis on the role of radiotherapy technique (IMRT vs 3D RT) in the PORT for NSCLC. They concluded that there was no correlation with toxicity in elderly patients, suggesting that the outcomes of PORT may be independent of the RT technique [15]. Nevertheless, our retrospective series contradicts these findings, as the toxicity outcomes were notably lower (toxicities ≥ G3: 2% for lung, 0,4% for heart) when compared to those documented in the LungART trial [1] (toxicities ≥ G3: 14% for lung, 5% for heart). Moreover, our results align closely with those of PORT-C [2] trial (toxicities ≥ G3: 0,7% for lung, 1,2% for heart), indicating a high degree of homogeneity, likely attributable to the use of more advanced RT technologies.

Regarding toxicity, a comprehensive study on different dosimetry parameters and dose constraints is essential to enhance the therapeutic ratio of modern RT. The safety and toxicity, particularly cardiopulmonary, remains a significant concern in relation to PORT, especially considering that some studies have not demonstrated a clear oncologic benefit. The excessive toxicity observed in patients receiving PORT in earlier trials, which led to non-cancer-related deaths, can be attributed to several factors such as suboptimal technique, large or excessive radiation volumes and the lack of CT-based planning. Several studies have investigated the role of modern radiation techniques with the mortality, suggesting that PORT does not impact the risk of death [16–18]. In this regard, a SEER analysis of PORT demonstrated that this technique was associated with an increased number of deaths from heart disease in patients between 1983 and 1988, but not in more recent cohorts [19]. The transition from conventional 2D to 3D radiation planning, and the subsequent advancements to 3D-CRT, IMRT, and VMAT, has significantly reduced radiation toxicity [20]. However, a dosimetric comparison of PORT plans using different techniques for pN2 NSCLC patients did not show absolute dosimetric advantages for all patients. Therefore, the choice of radiation therapy technique should be personalized, balancing target coverage with the protection of organs at risk.

Proton therapy offers a promising approach to reducing RT toxicity, with small series showing lower RT doses to heart and lungs [21, 22]. Boyce-Fappiano et al. found in a retrospective analysis proton beam PORT was associated with longer overall survival (OS) [22], due a better sparing of heart and lungs. Similarly, studies by Ma et al. [23] and Shepherd et al. [24] examined the correlation between dosimetry distribution to the heart and lungs and OS in patients treated with PORT. Ma et al. found that a higher dose to the heart and lungs was associated with a lower OS [23]. These findings align with the results from Shepherd et al., who reported a correlation between heart V8 and OS in PORT patients [24].

In this context, the results of our retrospective study provide valuable insights into the demographics and treatment characteristics, toxicity profiles, and outcomes of a cohort of 255 NSCLC patients treated with PORT with advanced RT techniques.

It’s also noteworthy to underline that the Impower010 trial has significantly impacted the landscape of resected stage III NSCLC by demonstrating the efficacy of adjuvant atezolizumab after chemotherapy, as evidenced by both disease free survival (DFS) [25] and OS (trend) [26]. Ongoing phase III clinical trials, such as BR.31 (durvalumab versus placebo) (NCT02273375), PEARLS/KEYNOTE-091 (pembrolizumab versus placebo) [27], ANVIL (nivolumab) [28], and ALCHEMIST [29], are investigating adjuvant immunotherapy in resected stage IB-IIIA NSCLC. Additionally, trials like Checkmate-816 [30], Keynote-671 [31], Checkmate-77t [32], Neotorch [33], and Aegean [34] are exploring neoadjuvant strategies combining chemotherapy and immunotherapy, showing promising results in obtaining complete pathological responses. This evolving landscape is expected to complicate the future management of resectable NSCLC in the near future.

Despite incorporating lower stages of NSCLC in these trials, the recurrence rate remains high, emphasizing the need for further investigation. Notably, in the IMpower010 trial, 29.8% of patients in the experimental arm experienced recurrence at 24 months, with disease relapse strongly correlated with OS in the immunotherapy arm [25]. Even though only 11% of patients in the experimental group and 16% in the observation group received subsequent salvage RT, details about the radiotherapy intent in this subgroup remain unclear [25].

Given this context, it is crucial to scrutinize the recurrence patterns in patients submitted to adjuvant combined systemic therapies and explore the different approaches within stratified risk classes. On the other hand, understanding whether immunotherapy may mitigate distant recurrence may also guide the consideration of local RT in select subsets of patients at high risk of local recurrence.

The demographic and clinical characteristics of the analyzed population reflect the heterogeneous nature of lung cancer patients and underline the importance of considering these factors in the whole treatment planning. It is noteworthy to underline that the decision regarding adjuvant chemotherapy was not uniform, with a notable subset of patients not receiving systemic therapy (24.7%). This variability may reflect individualized treatment decisions influenced by patient-specific factors and the evolving landscape of adjuvant therapies for lung cancer. Regarding RT, the low rate of temporary or definitive interruptions in our population confirmed the effective treatment planning and delivery with advanced techniques, minimizing disruptions that can affect treatment efficacy. Despite that, a significant proportion of patients experienced acute toxicities, with pulmonary and esophageal side effects being the most common, mostly mild to moderate. Notably, a small percentage of patients experienced acute cardiac toxicity, emphasizing the importance of cardiac sparing techniques during the radiotherapy planning. Chronic toxicities, though less frequent, highlight the actual need for long-term monitoring and management of treatment-related side effects.

The correlation analysis conducted in this study revealed several significant associations between clinical variables, dosimetry parameters and toxicity outcomes. Particularly, preexisting cardiac disease, the number of excised nodes and radiation dosimetry seem to play a significant role on influencing toxicity profiles. These findings also highlight the importance of personalizing each treatment plan considering patient-specific factors as well as minimizing the risk of treatment-related toxicities. In this scenario, the RT induced cardiac toxicity and coronary artery disease represent an important issue, as an important cause of mortality and morbidity in survivors p [35].

In conclusion, our study, as well as recent clinical trials, contributes to the evolving discussion on PORT inclusion criteria in resected stage III lung cancer. The interplay between historical data, contemporary trials, and our retrospective analysis offers a more comprehensive understanding of the complexities surrounding PORT in stage III resected NSCLC patients. While the debate continues, the emerging focus on factors such as diffuse use of advanced RT techniques, new dose constraints and novel therapeutic strategies may help to refine the selection of patients who stand to benefit most from PORT. Personalized decisions on treatment planning should always consider the broader context of each patient's unique characteristics with the ultimate goal of optimizing outcomes and enhancing the care of lung cancer patients. Further research and validation are still warranted to advance our knowledge and improve the management of this challenging disease.
